# Methyl jasmonate, salicylic acid, and oxalic acid affects growth, inducible defenses, and pine weevil resistance in Norway spruce

**DOI:** 10.3389/fpls.2023.1155170

**Published:** 2023-07-06

**Authors:** Paal Krokene, Ketil Kohmann, Ngan Bao Huynh, Melissa H. Mageroy

**Affiliations:** ^1^Division of Biotechnology and Plant Health, Norwegian Institute of Bioeconomy Research, Ås, Norway; ^2^Division of Forest and Forest Resources, Norwegian Institute of Bioeconomy Research, Ås, Norway

**Keywords:** methyl jasmonate (MeJA), oxalic acid, *Picea abies* (L) Karst., *Hylobius abietis*, salicylic acid, traumatic resin ducts

## Abstract

The large pine weevil (*Hylobius abietis*) is a major regeneration pest in commercial forestry. Pesticide application has historically been the preferred control method, but pesticides are now being phased out in several countries for environmental reasons. There is, thus, a need for alternative plant protection strategies. We applied methyl jasmonate (MeJA), salicylic acid (SA) or oxalic acid (OxA) on the stem of 2-year-old Norway spruce (*Picea abies*) plants to determine effects on inducible defenses and plant growth. Anatomical examination of stem cross-sections 9 weeks after application of 100 mM MeJA revealed massive formation of traumatic resin ducts and greatly reduced sapwood growth. Application of high concentrations of SA or OxA (500 and 200 mM, respectively) induced much weaker physiological responses than 100 mM MeJA. All three treatments reduced plant height growth significantly, but the reduction was larger for MeJA (~55%) than for SA and OxA (34-35%). Lower MeJA concentrations (5-50 mM) induced comparable traumatic resin duct formation as the high MeJA concentration but caused moderate (and non-significant) reductions in plant growth. Two-year-old spruce plants treated with 100 mM MeJA showed reduced mortality after exposure to pine weevils in the field, and this enhanced resistance-effect was statistically significant for three years after treatment.

## Introduction

Norway spruce (*Picea abies*) and Scots pine (*Pinus sylvestris*) are economically important and ecologically dominant tree species in Europe’s boreal and subalpine forests (Buras and Menzel, 2019). A major challenge to reforestation of these species after harvesting is attack by the large pine weevil (*Hylobius abietis*), a regeneration pest that may girdle and kill young plants (Nilsson et al., 2010). As Norway spruce forests usually are regenerated by planting nursery-grown plants after clear-cutting, pine weevil impacts are most severe in spruce forests. If no preventive measures are taken, pine weevil feeding may kill 60% or more of newly planted spruce trees (Örlander and Nilsson, 1999). Historically, insecticides such as DDT, lindane, and permethrin were used to protect young spruce plants from pine weevil attack (Nilsson et al., 2010). However, the use of insecticides is now being banned or phased out in many countries due to environmental concerns (Nilsson et al., 2010). In their place, integrated pest management incorporating soil scarification, physical stem protection, and other measures may be effective in reducing pine weevil damage (Dillon and Griffin, 2008; Galko et al., 2022). Yet, another and perhaps more cost-effective protection measure could be to stimulate the trees’ own defenses using defense elicitors.

Norway spruce has multiple constitutive and inducible defenses against pests and pathogens. Constitutive defenses are always present and provide the first line of protection (Franceschi et al., 2005). These defenses include physical barriers, such as cork bark and other tissues reinforced by lignin and suberin, as well as chemical defenses such as terpenes stored in preformed resin ducts and phenolic compounds stored in polyphenolic parenchyma (PP) cells (Krokene, 2015). Upon attack, injury or other stresses, Norway spruce activates inducible defenses for enhanced protection. Inducible defenses include increased resin production in preformed resin ducts, formation of novel traumatic resin ducts (Nagy et al., 2000), and activation of PP cells (Franceschi et al., 1998; Krokene et al., 2008a). Inducible defenses can be activated immediately upon attack or be delayed/primed, whereby a plant sensitized by previous stress responds quicker or more strongly to a subsequent attack (Conrath et al., 2015; Mageroy et al., 2020a).

Many studies have shown that exogenous application of the phytohormone methyl jasmonate (MeJA) triggers inducible resistance in Norway spruce against fungal infection and insect attack through a combination of direct activation and priming of tree defenses (Martin et al., 2002; Erbilgin et al., 2006; Krokene et al., 2008b; Mageroy et al., 2020b). However, using MeJA to boost tree resistance can also have negative effects such as reducing height growth (Heijari et al., 2005; Moreira et al., 2012; Zas et al., 2014), reducing sapwood growth, reducing tracheid cell lumen area, reducing net photosynthetic rate or causing stomatal closure (Heijari et al., 2005). The magnitude of these negative effects varies among studies and with the concentration of MeJA used, suggesting that optimal usage of MeJA for practical tree protection requires knowledge about the costs and benefits associated with different dosages. Additionally, there could be other chemical priming stimuli that increase tree resistance with less negative effects.

Salicylic acid (SA) and oxalic acid (OxA) are two candidate chemicals that are known to elicit inducible defenses in conifers. SA, a plant hormone with a key regulatory role in inducible defenses, has been shown to accumulate after pathogen infection in Norway spruce (Kozlowski and Metraux, 1998; Kozlowski et al., 1999; Likar and Regvar, 2008). Exogenous application of SA on Norway spruce can reduce colonization by the spruce bark beetle *Ips typographus* (Urbanek Krajnc et al., 2011; Felicijan et al., 2016). Treatment with SA also increases resin production in *Pinus pinaster* (Michavila Puente-Villegas et al., 2021) and *Pinus elliotti* (Rodrigues and Fett-Neto, 2009). OxA is a pathogenicity factor that enables several phytopathogenic fungi to infect their host (Ikotun, 1984; Kumari et al., 2014), but it can also directly trigger systemic resistance in plants (Doubrava et al., 1988; Singh et al., 2002; Zhang et al., 2015). Krokene et al. (2008b) found that exogenous application of OxA reduced symptoms of fungal infection in 13-year-old Norway spruce trees.

In this study, we assessed effects of exogenous application of OxA, SA, and MeJA on growth, defense responses, and resistance of 2-year-old Norway spruce plants. We also did a dose-response experiment with MeJA, the most commonly used chemical priming stimulus in Norway spruce, to test how different MeJA concentrations affects plant growth and defenses. Finally, we tested if MeJA treatment increased plant resistance to pine weevil attacks over three growing seasons in a field experiment.

## Materials and methods

### Treatment of plants with MeJA, SA or OxA

Two-year-old Norway spruce plants were treated with methyl jasmonate (MeJA), salicylic acid (SA) or oxalic acid (OxA) to study effects on growth and inducible defense responses. Containerized plants from two different full-sib families were obtained from a commercial nursery. Two different families were used because the nursery could not provide enough plants from a single family for two different experiments. Seedlings were potted in 8 cm pots on 5-6 June 2001 and placed in a greenhouse. When all plants had broken bud and started shoot elongation (21 June) they were treated with chemicals by carefully coating the first stem internode using a small, soft paintbrush. In experiment 1, 60 plants from one family were randomly allocated to treatment with 100 mM MeJA (in water with 0.1% Tween 20), 200 mM OxA (in water), 500 mM SA (in 60% ethanol), water with 0.1% Tween 20 or no treatment (“MeJA-OxA-SA experiment”; n = 12). Tween 20 was used to solubilize MeJA and to act as a surfactant to help spread the solution evenly on the hydrophobic bark surface (Franceschi et al., 2002). In experiment 2, 60 plants from the other family were randomly allocated to a dose-response experiment with MeJA (0, 5, 25, 50 or 100 mM MeJA in water with 0.1% Tween 20) (“MeJA dose-response experiment”; n = 12). For an overview of the experimental design, tree measurements and number of replicates, see **Supplemental Figure 1**.

### Fungal inoculation of plants

About four weeks after chemical treatment (18 July), when the length of the apical shoot averaged 55 mm, plants were inoculated with a pathogenic fungus to test whether treatment with SA, OxA or different doses of MeJA affected plant resistance. Nine plants per treatment were inoculated with the pathogenic bluestain fungus *Endoconidiophora polonica* (isolate 93-208/115 from the culture collection of the Norwegian Institute of Bioeconomy Research) and three plants were left intact as uninoculated controls. This fungus is a necrotrophic pathogen vectored mainly by the spruce bark beetle *Ips typographus* (Kirisits, 2004). Normally, *E. polonica* infects the phloem and sapwood of mature Norway spruce trees that have been attacked by *I. typographus* but the fungus will also infect younger trees/plants if inoculated experimentally. It has therefore been used as a tool to induce tree defenses in several previous studies of resistance mechanisms in Norway spruce (e.g. Krokene et al. 2008b; Fossdal et al., 2012; Zhao et al., 2019). Plants were inoculated by incising a ~5 mm wide cut into the stem bark, extending around half the stem circumference midway up on the first internode. Inoculum, consisting of actively growing mycelium of fungus on malt agar (2% malt, 1.5% agar), was placed beneath the bark, and parafilm was wrapped around the inoculation wound to seal the bark back to the stem and to prevent contamination and excessive drying of phloem and sapwood. Five weeks after inoculation (22-23 August), stem cross-sections were cut from six inoculated plants per treatment and examined for symptoms of fungal infection under a Leitz stereomicroscope (Ernst Leitz GMBH, Wetzlar, Germany) (**Supplemental Figure 1**).

### Stem anatomy and plant growth

Plants were harvested when they had completed shoot growth (22-23 August), ~5 weeks after fungal inoculation and ~9 weeks after chemical treatment. Stem tissues for anatomical studies were collected from three inoculated and three intact plants per treatment (**Supplemental Figure 1**). A stem section extending from the inoculation site and ~5 mm downwards was cut from each plant using a razor blade. The 5-mm stem sections were placed directly in fresh fixative (2% paraformaldehyde and 1.25% glutaraldehyde buffered in 50 mM L-piperazine-N-N’-bis (2-ethane sulfonic) acid, pH 7.2) and stored until further processing. Semi-thin sections (15 µm thick), including the whole cross-sectional stem area, were cut near the inoculation site using a cryotome (Cryo-Star HM 560, Microm International GmbH, Walldorf, Germany). Stem cross-sections were stained with Stevenel’s blue (del Cerro et al., 1980) and mounted with immersion oil for analysis of general anatomy, parenchyma cells, polyphenolic inclusions within these cells, and traumatic resin ducts (Nagy et al., 2000).

On each stem cross-section we measured total area of bark (i.e., all tissues outside the vascular cambium) and sapwood (both current-year sapwood and total sapwood) at 32× magnification using a Leitz stereomicroscope (Ernst Leitz GMBH, Wetzlar, Germany). We also measured the combined cross-sectional area of all resin ducts present in the sapwood and of cortical resin ducts in the phloem. On sapwood cross-sections, imaged at 5 or 10× using a Leitz Aristoplan light microscope (Ernst Leitz GMBH, Wetzlar, Germany) and bright-field optics, we counted the total number of tracheid cells in the current-year sapwood along three radial cell files per cross-section. If traumatic resin ducts were present, we also counted the number of tracheids laid down in the earlywood, before the traumatic resin ducts were formed. On phloem cross-sections imaged at 10× using the light microscope we measured the combined area of all polyphenolic inclusions inside parenchyma cells inside a randomly selected section of the secondary phloem. All cross-sections were imaged using a Leica DC200 or DC300 CCD camera (Leica Microscopy Systems Ltd., Heerbrugg) and anatomical structures were quantified as described in Nagy et al. (2004).

Height growth of the plants was determined by measuring the length of the apical shoot at the time of inoculation (18 July) and at harvesting, 5 weeks later (22-23 August) (**Supplemental Figure 1**). Height growth was calculated as the difference between the two measurements.

### Plant resistance to the pine weevil *Hylobius abietis*

In a third experiment, two-year-old Norway spruce plants were obtained from a commercial nursery in October 2000 and cold-stored until spring 2001. On 14 June 2001, plants were planted out in a fresh clear-cut in Kjølstad, Ås, SE Norway. Before planting, groups of 30 plants were subjected to four different treatments: (1) treatment with 100 mM MeJA (in water with 0.1% Tween 20) on 11 May using the same procedure as for the greenhouse-grown plants, (2) spray application with the pesticide Gori 920 LX (active ingredient: permethrin, 235 gL^-1^) on 13 June as a positive control (this pesticide was the industry standard for chemical protection of young spruce plants in Norway at the time of the experiment), (3) mechanical wounding by puncturing the bark of the lower stem using a metal brush, or (4) no treatment (negative control). Spray application of the pesticide was done outdoors and away from the other plants, to prevent contamination of unsprayed plants. Plants were planted in a randomised complete block design, with 30 blocks containing one plant subjected to each of the four treatments (120 plants in total). On the clear-cut, plants were exposed to natural attacks of the pine weevil *Hylobius abietis* for three consecutive growing seasons. Each autumn, we recorded the number of plants that had been girdled and killed by the beetles.

### Data treatment and statistical analysis

Statistical analyses were performed using RStudio Server (v2022.02.2 Build 485; R Core Team, 2022) and the packages emmeans (v1.7.3; Lenth et al., 2022), lme4 (Bates et al., 2015), mixlm (v1.2.6; Liland, 2022), rstatix (v0.7.0; Kassambara, 2021), and stats (v4.2.0; R Core Team, 2022). All anatomical parameters were fitted with a linear model (estimated using Ordinary Least Squares) to predict the effect of treatment on anatomical features (formula: feature ~ treatment + incoulation). We obtained linear models with a non-significant main effect of “inoculation” and a non-significant interaction term between “treatment” and “inoculation”. Therefore, we combined data from inoculated and uninoculated plants for analyses of anatomical parameters and height growth to not overfit the model. Statistical significance was determined using a one-way ANOVA (formula: feature ~ treatment) with Tukey’s HSD *post-hoc* testing. Because there were no statistical significant differences between the two control treatments used in the MeJA-OxA-SA experiment (water with 0.1% Tween or no treatment) we combined these two controls in the statistical analyses. Data on plant survival following weevil exposure was fitted with a logistic mixed model (estimated using Maximum Likelihood and Nelder-Mead optimizer) to predict the effect of treatment on survival status (dead or alive) (formula: survival status ~ treatment). The model included year and tree ID as random effects [formula: ~ treatment + (1|year)]. Analysis of deviance of survival data was performing using Type II Wald Chi-Square tests. Pairwise statistical significance was determined using Tukey’s HSD *post-hoc* testing. Figures were generated using ggfortify (Tang et al., 2016), ggplot2 (Wickham, 2016), ggpubr (v0.4.0; Kassambara, 2020), and RColorBrewer (v1.1-3, Neuwirth, 2022).

## Results

### Effects of MeJA, SA, and OxA on plant growth

**Figure 1:**
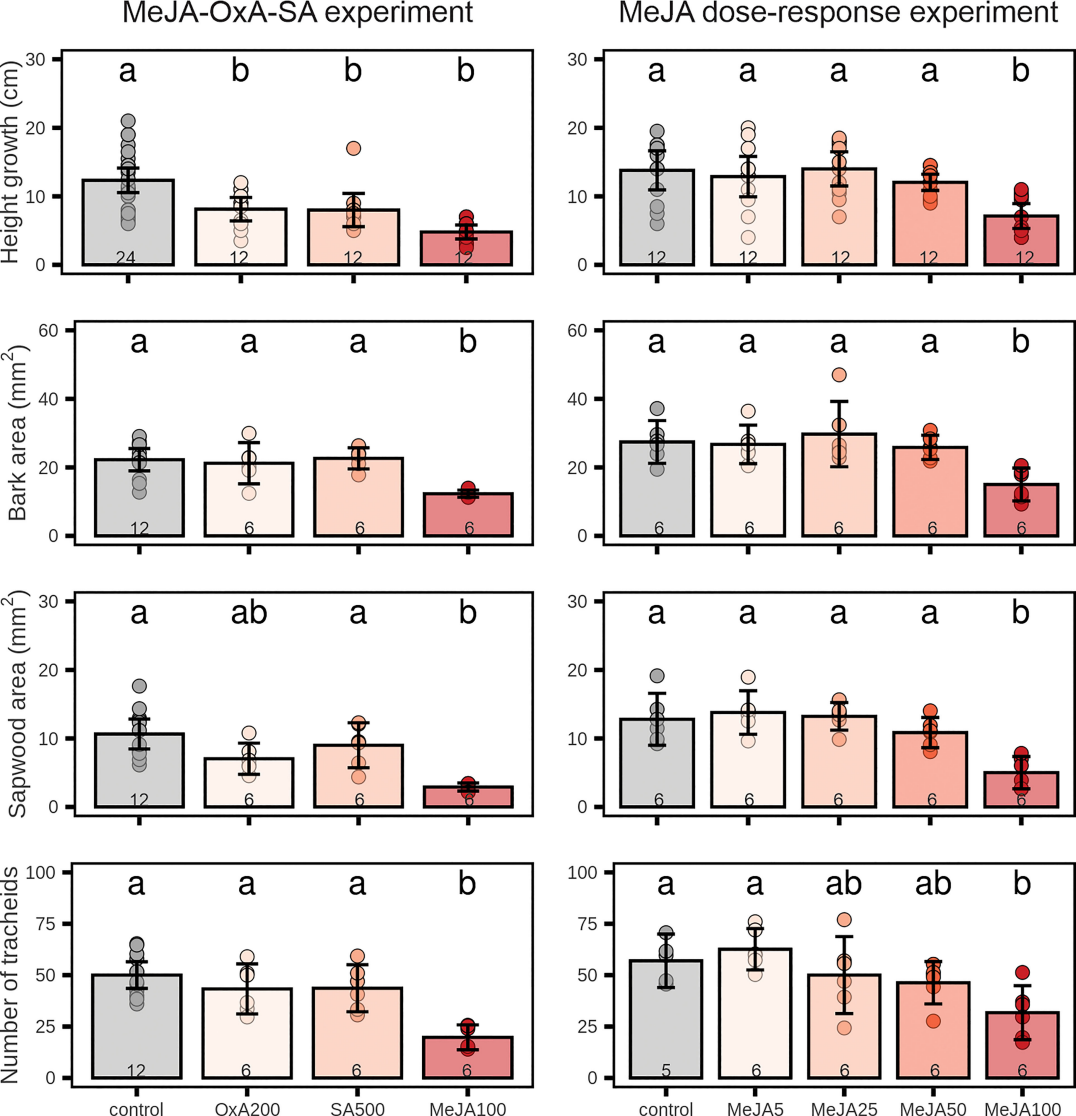
Growth in 2-year-old Norway spruce plants after application of different chemicals on the stem bark. Left panels: treatment with high concentrations of salicylic acid (SA; 500 mM), oxalic acid (OxA; 200 mM) and methyl jasmonate (MeJA; 100 mM). Right panels: treatment with different MeJA concentrations (0, 5, 25, 50, 100 mM). Height growth was measured over a 9-week period after chemical treatment. Current-year sapwood area, total bark area, and tracheid numbers in the current-year sapwood were determined on stem cross-sections sampled 9 weeks after chemical treatment and visualized using a microscope. Bars represent treatment group means ± 95% confidence interval of the mean. Points represent individual replicates. Number inside the bars indicate the sample size (n). Treatment groups which do not share the same letter are significantly different (1-way ANOVA (~ treatment) followed by Tukey *post-hoc* test, p < 0.05).

**Figure 2:**
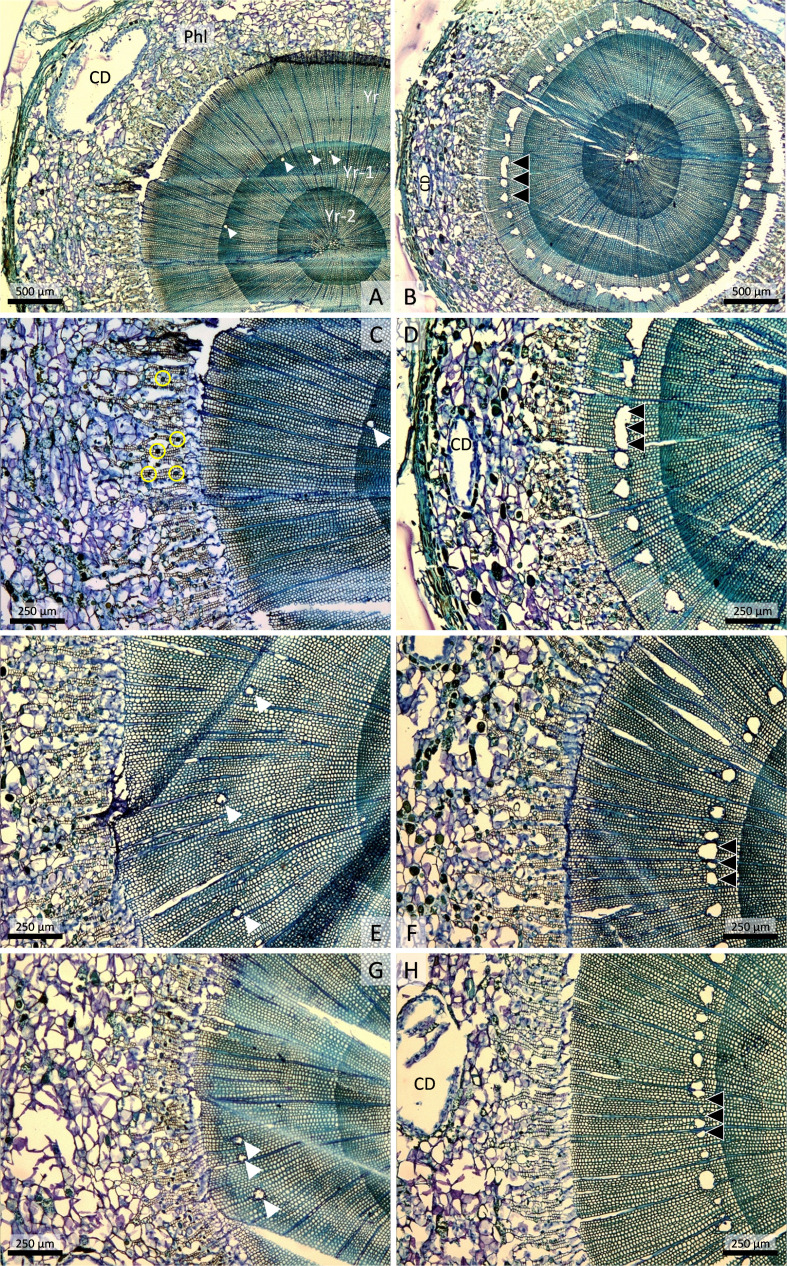
Stem cross-sections of 2-year-old Norway spruce plants collected 9 weeks after the start of the experiment. **(A)** Untreated control plant with phloem (Phl), containing a large cortical resin duct (CD), and three annual rings of xylem growth (Yr, Yr-1, Yr-2). Scattered resin ducts (white arrowheads) are visible in the Yr-1 annual ring (Yr-1). **(B)** Plant treated with 100 mM methyl jasmonate (MeJA) 9 weeks earlier. An almost continuous ring of traumatic resin ducts (triple black arrowheads) is visible in the current annual xylem growth, which is much narrower than in the control. **(C)** Higher magnification of A showing sieve cells interspersed with rounded parenchyma cells with vacuolar polyphenolic inclusions in the phloem (small yellow circles). **(D)** Higher magnification of B showing traumatic resin ducts with very wide lumens and coalescence of adjoining ducts. **(E)** Untreated control plant that was inoculated with the fungus *Endoconidiophora polonica* 4 weeks after the start of the experiment. **(F)** Plant treated with 50 mM MeJA 9 weeks earlier has numerous traumatic resin ducts and normal annual xylem growth. **(G)** Plant treated with 200 mM oxalic acid 9 weeks earlier. **(H)** Plant treated with 5 mM MeJA 9 weeks earlier has extensive traumatic resin duct formation and normal annual xylem growth.

High concentrations of MeJA, SA or OxA reduced height growth significantly relative to the controls, with MeJA having the strongest effect (**Figure 1**). MeJA (but not OxA or SA) also inhibited xylem and phloem growth (**Figures 1**, **2**). Growth reduction for the 100 mM MeJA treatment was greater in the MeJA-OxA-SA experiment (61% reduction relative to the control) than in the MeJA dose-response experiment (48% reduction). Trees treated with 100 mM MeJA had smaller phloem cross-sectional area than the controls (45% in both experiments), smaller current-year sapwood area (73% in MeJA-OxA-SA experiment, 61% in MeJA dose-response experiment), and laid down fewer tracheids in the current annual ring (60% in MeJA-OxA-SA experiment, 44% in MeJA dose-response experiment) (**Figure 1**). Lower concentrations of MeJA had small and non-significant effects on growth (**Figure 1**). OxA and SA reduced height growth significantly (by 34 and 35%, respectively) but had no significant effect on phloem area, sapwood area or tracheid numbers (**Figure 1**).

### Plant resistance to fungal inoculation

To evaluate effects of SA, OxA and different doses of MeJA on induced resistance, plants were inoculated with the bluestain fungus *E. polonica* or left intact. Inoculated plants showed mild and inconsistent symptoms five weeks after inoculation. Symptomatic plants had some necrotic phloem near the inoculation site, but the necrotic area varied both within and between treatments and there were no consistent differences between control plants (not treated with chemicals) and plants treated with SA, OxA or different doses of MeJA.

### Anatomical responses to MeJA, SA, and OxA

In stem cross-sections of untreated control plants, we observed phloem, mostly consisting of cortex, and three annual rings of xylem/sapwood growth (**Figure 2A**). The cortex, which represents primary growth, consisted of sieve cells, rounded parenchyma cells with polyphenolic vacuolar inclusions, and some large cortical resin ducts (**Figures 2A, C**). Only occasional axial resin ducts were observed in the last two annual layers of sapwood growth in the controls (**Figures 2A, C, E**). In plants treated with 100 mM MeJA, the surface areas of phloem and current-year xylem growth were smaller than in the control, and a nearly continuous ring of traumatic resin ducts was present (**Figures 2B, D**). Extensive traumatic resin ducts were also present in the current annual xylem growth in plants treated with lower concentrations of MeJA (**Figures 2F, H**). The traumatic resin ducts had wider lumens than regular axial resin ducts, and neighboring ducts had sometimes coalesced to form very wide resin ducts (**Figures 2B, D**). Plants treated with lower concentrations of MeJA had normal xylem growth similar to untreated controls (**Figures 2F, H**). Occasional axial resin ducts were also found in the xylem of control plants and plants treated with OxA, but these ducts always occurred further out in the annual ring (**Figures 2E, G**) and were probably induced by wounding associated with fungal inoculation (occurring 4 weeks after elicitor treatment).

**Figure 3:**
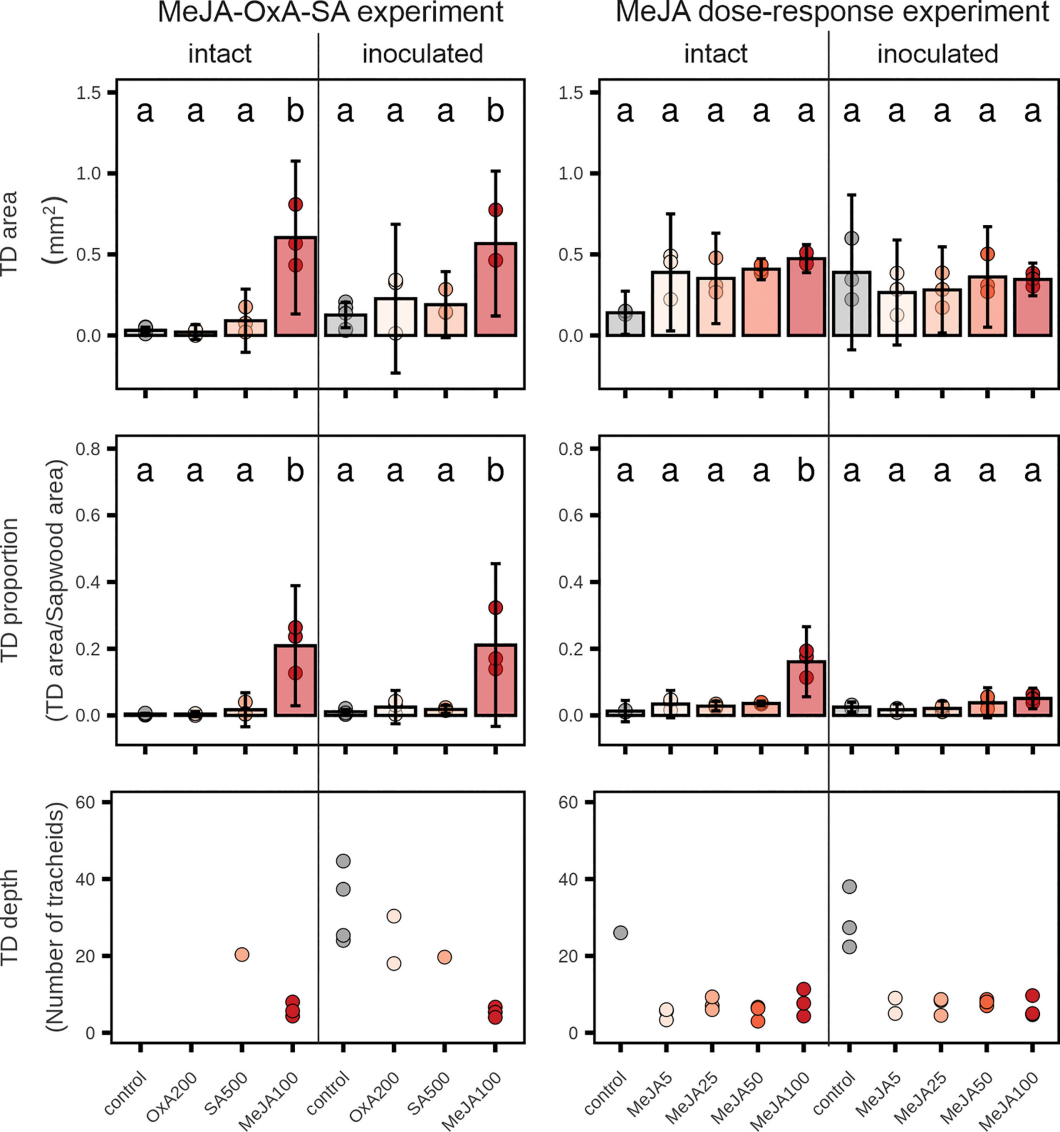
Traumatic resin duct (TD) formation in 2-year-old Norway spruce plants 9 weeks after application of different chemicals on the stem bark. Four weeks after application, three plants per treatment were inoculated with the fungus *Endoconidiophora polonica* and three plants were left intact. Upper row: cross-sectional TD area after treatment with high concentrations of oxalic acid (OxA; 200 mM), salicylic acid (SA; 500 mM) or methyl jasmonate (MeJA; 100 mM) or different concentrations of MeJA (0, 5, 25, 50 or 100 mM with 0.1% Tween 20). Control plants in the MeJA-OxA-SA experiment were untreated or were treated with 0.1% Tween 20 (n = 3 + 3 = 6). Control plants in MeJA dose-response experiment were treated with 0.1% Tween 20 (n = 3). Middle row: cross-sectional TD area relative to the total sapwood area of the current annual ring. Lower row: the number of tracheids laid down in the xylem before the TDs were formed (only plants with resin duct formation are included; n = 1-4). Bars represent treatment group means ± 95% confidence interval of the mean. Points represent individual replicates. Treatment groups that do not share the same letter are significantly different (1-way ANOVA (~ treatment) followed by Tukey *post-hoc*, p < 0.05).

The total area of traumatic resin ducts was significantly larger in plants treated with 100 mM MeJA in the MeJA-OxA-SA experiment than in the other treatments (**Figure 3**). This was the case both in intact plants and in plants that had been inoculated with *E. polonica* (**Figure 3**). In the MeJA dose-response experiment, there were no significant differences in traumatic resin duct area between control and MeJA treatments. However, in uninoculated plants resin duct areas tended to be greater for all MeJA dosages compared to controls (**Figure 3**).

The proportion of total current-year sapwood area that was made up by traumatic resin ducts in the MeJA-OxA-SA experiment was significantly larger in plants treated with 100 mM MeJA than in the other treatments. The traumatic resin duct proportion was also significantly higher in plants treated with 100 mM MeJA than in other treatments in the MeJA dose-response experiment, but only in uninoculated plants (**Figure 3**).

The number of tracheids produced in the current-year sapwood before the traumatic resin ducts appeared was always lower in MeJA-treated plants than in control plants and plants treated with OxA or SA (**Figure 3**). The difference between MeJA-treated plants and non-MeJA-treated plants amounted to about 10-35 tracheids. No significant effects of OxA, SA or MeJA were observed on the size of cortical resin ducts or area of polyphenolic inclusions inside PP cells (**Supplemental Figure S2**).

### Effect of methyl jasmonate on weevil damage

**Figure 4:**
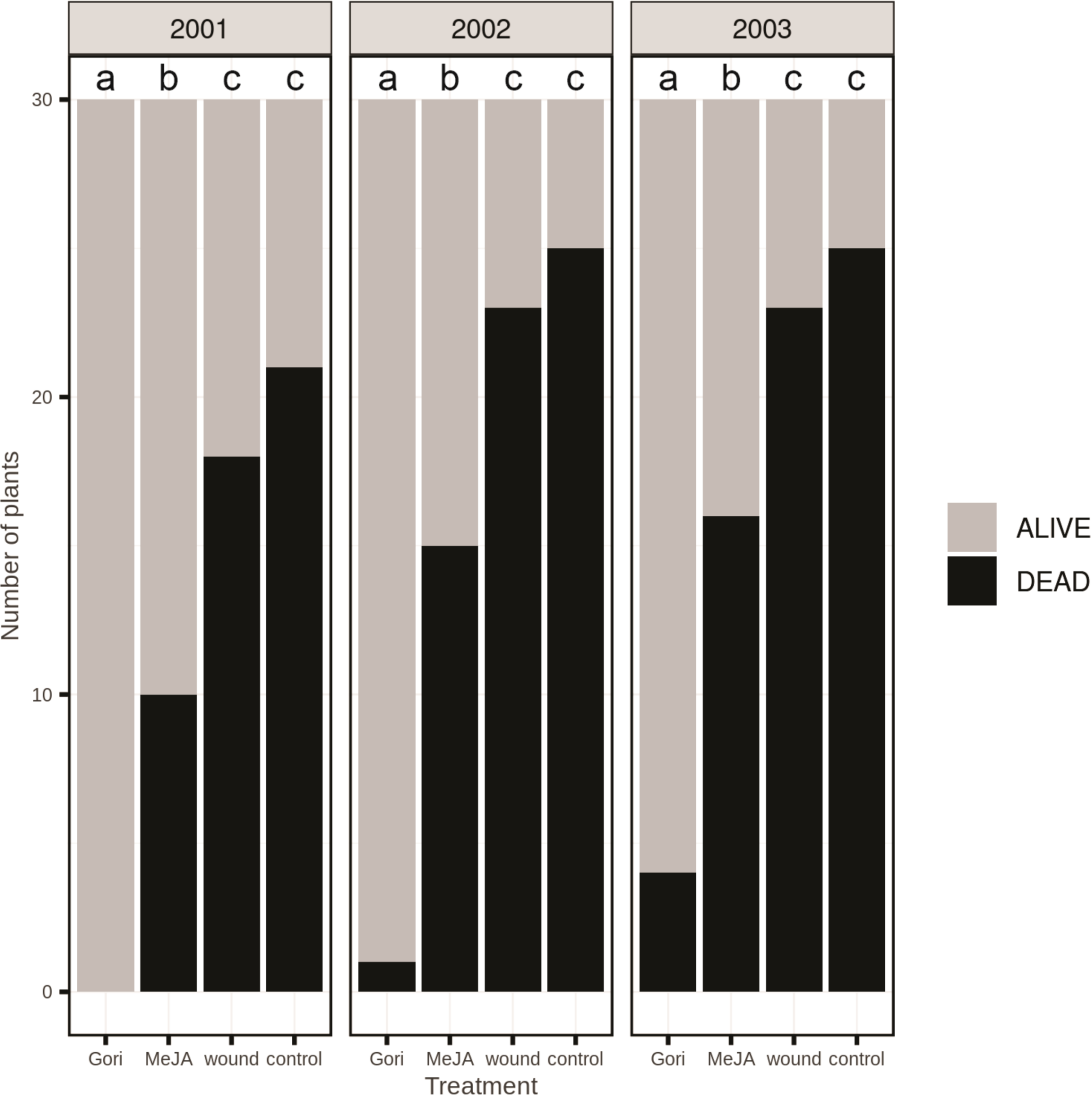
Accumulated pine weevil-inflicted mortality of 2-year-old Norway spruce plants over three growing seasons following planting in the field. Shortly before planting, the stem of the plants was treated with the pyrethroid Gori 920 LX (Gori), 100 mM methyl jasmonate (MeJA), mechanical wounding (wound) or remained untreated as a control. Black bars = dead plants that had been completely girdled by the weevils; grey bars = plants with no or moderate weevil damage. n = 30 plants per treatment. Treatment groups that do not share the same letter are significantly different (Wald Chi square [~ treatment + (1|year)] followed by Tukey *post-hoc*, p < 0.05).

Treatment with MeJA reduced pine weevil damage considerably the first growing season, when plant mortality was only 33%, compared to 60% mortality in wounded plants and 70% in untreated control plants (**Figure 4**). Pesticide treatment gave complete protection the first season (no plant mortality). In the second year, mortality increased somewhat in MeJA-treated, wounded, and control plants, and accumulated mortality reached 50, 77, and 83%, respectively. Only one pesticide-treated plant died during the second year (3% accumulated mortality). In the third year, very few additional plants died in the different treatments, except for pesticide-treated plants where accumulated mortality reached 13%. Overall, pesticide treatment was most effective in reducing mortality due to pine weevil attack (χ^2^ (3, N = 120) = 39.9, *p* < 0.001). MeJA was significantly more effective than both control and wounding (*p* < 0.001), but less effective than the pesticide (*p* < 0.001). There was no significant difference between wounding and the untreated control (*p* = 0.62).

## Discussion

The use of natural compounds to enhance plant resistance to pests and diseases is an attractive alternative crop protection concept to pesticide/fungicide application (Aranega-Bou et al., 2014; Cooper and Ton, 2022). In conifers, methyl jasmonate (MeJA) has been used for more than 20 years to study inducible defenses, including defense priming, mostly in spruce (*Picea* spp.) and pine (*Pinus* spp.) (Kozlowski and Metraux, 1998; Martin et al., 2002; Miller et al., 2005; Zeneli et al., 2006; Zulak and Bohlmann, 2010; Celedon et al., 2017; Mageroy et al., 2020a; Chen et al., 2021; Puentes et al., 2021). In this study, we quantified inducible defense responses in 2-year-old Norway spruce plants following stem application of oxalic acid (OxA), salicylic acid (SA), and MeJA. We found that MeJA induced much stronger defense responses than OxA and SA: plants treated with MeJA formed an almost continuous ring of traumatic resin ducts around the stem circumference, whereas traumatic resin ducts formed only sporadically in plants treated with OxA or SA (**Figure 2**). Previously, OxA has been shown to reduce disease symptoms in 13-year-old Norway spruce trees infected with a fungal pathogen, but to a much smaller extent than MeJA (Krokene et al., 2008b). SA has been found to reduce bark beetle colonization of mature Norway spruce trees when applied on the stem bark prior to beetle attack (Urbanek Krajnc et al., 2011; Felicijan et al., 2016). This plant hormone is an important activator of genes encoding pathogenesis-related proteins (Durner et al., 1997), i.e., proteins induced in response to pathogen infection (van Loon, 1997). SA accumulates in response to pathogen infection and MeJA treatment in spruce (Kozlowski and Metraux, 1998; Kozlowski et al., 1999) but there is still no evidence that SA induces conifer defense responses *in planta* and any mechanisms of SA-induced resistance in conifers remain unknown.

Unfortunately, we were unable to determine whether treatment with MeJA, SA or OxA increased plant resistance to fungal infection in this experiment, as the fungus failed to infect either chemically treated plants or control plants. The fungal isolate we used has been shown to be pathogenic to both 2-year-old and mature Norway spruce trees in previous studies (e.g., Krokene and Solheim, 1998; Krokene et al., 1999; Krokene et al., 2001). The restricted symptoms induced in the present study suggest that the inoculation load we used was too small relative to the resistance of the plants. In previous studies, MeJA treatment has been shown to increase resistance to fungal infection in Norway spruce. Stem treatment of mature Norway spruce trees with 5 to 100 mM MeJA reduced symptoms of fungal infection in a dose-dependent manner (Zeneli et al., 2006). Additionally, foliar treatment of 2-year-old plants with 10 mM MeJA reduced stem colonization of the bluestain fungus *Grosmannia penicillata* (Wilkinson et al., 2022). These previous studies demonstrate that even low MeJA concentrations can induce pathogen resistance in Norway spruce.

Application of high MeJA concentrations (100 mM) lead to massive formation of traumatic resin ducts and up to 72% reduced height or stem growth in Norway spruce plants (**Figures 1**, **3**). Reduced growth following MeJA application has also been observed in previous studies and could be due to trade-offs between growth and defense (Heijari et al., 2005; Krokene et al., 2008b; Chen et al., 2021). We observed no toxic effects of MeJA, as the plants continued to grow for 9 weeks after MeJA application without browning, needle loss, or other visible signs of damage. Effects of MeJA on tree defenses and growth appeared to be genetically controlled, as treatment with 100 mM MeJA had more pronounced effects on growth reduction and traumatic resin duct formation in the family used in the MeJA-SA-OxA experiment than in the family used in the MeJA dose experiment (**Figures 2**, **4**). Genotype-specific responses to MeJA-treatment have previously been observed by Zeneli et al. (2006) in mature, clonal Norway spruce trees: some clones had little or no response to MeJA, whereas others responded with extensive traumatic resin duct formation. Understanding the genetic and molecular mechanisms underlying this differential response would be of interest in future research.

Interestingly, low MeJA concentrations (5-50 mM) induced comparable traumatic resin duct formation as 100 mM MeJA but without any obvious negative effects on growth (**Figures 1**–**3**). In terms of absolute traumatic resin duct area, plants treated with low MeJA concentrations had 2-3 times more resin ducts than control plants but did not differ significantly from controls (probably due to the low number of replicates; n = 3). Stem cross-sectioning confirmed that even the lowest MeJA concentration we tested (5 mM) induced extensive traumatic resin duct formation (**Figure 2H**). Thus, low MeJA concentrations did induce defense responses (and, by extension, probably also resistance) in young Norway spruce plants. The fact that low MeJA concentrations elicited strong inducible defense responses with minimal negative effects on growth suggests that low concentrations may be optimal for practical tree protection, e.g., in tree nursery production. Chen et al. (2021) showed that low MeJA concentrations (10 mM) significantly reduced pine weevil feeding on 1- and 2-year-old Norway spruce plants when plants were exposed to weevil feeding for 48 hours in lab bioassays. In the field, treatment with 10 mM MeJA reduced weevil feeding by about 80% 3 and 12 months after planting but the effect was not statistically significant. The 10 mM treatment also reduced plant height growth significantly 3 and 12 months after planting (Chen et al., 2021). Thus, effects of low MeJA concentrations on growth and resistance of young Norway spruce plants appear to be variable and to depend on experimental conditions. More studies are therefore needed before optimal MeJA concentrations for robust operational plant protection in forest nurseries can be suggested.

Both MeJA treatment and inoculation/wounding induced traumatic resin duct formation in our study. The anatomical analyses suggested that in non-MeJA-treated plants traumatic resin ducts were mainly formed in response to wounding incurred by the inoculation process, as the fungus did not successfully colonize the stem. Formation of traumatic resin ducts in response to wounding has been observed in previous studies (Nagy et al., 2000; Byun-McKay et al., 2006). In our study, traumatic resin ducts in non-MeJA-treated plants occurred further out in the current-year sapwood growth compared to MeJA-treated plants, i.e., they were produced later in the season. The 10-35 additional tracheids that were produced prior to traumatic resin duct formation in non-MeJA-treated compared to MeJA-treated plants (**Figure 3**) correspond roughly to the number of tracheids Norway spruce trees produce in a 4-week period [the interval between chemical treatment (21 June) and fungal inoculation (18 July); Krekling et al., 2004]. Traumatic resin duct formation was always more extensive in MeJA-treated plants than in non-MeJA-treated plants (although not always significantly so). The ducts also occurred deeper in the current-year sapwood in MeJA-treated plants (**Figure 3**), indicating that they were induced earlier in the growing season. There were no clear differences in absolute traumatic resin duct area between MeJA concentrations, but the ducts made up a much larger proportion of the sapwood area in plants treated with the highest MeJA concentration (100 mM). This is simply because the highest MeJA concentration reduced sapwood growth several-fold compared to lower MeJA concentrations (**Figures 2B, F, H**).

In our field experiment we showed that treatment with 100 mM MeJA reduced pine weevil damage in 2-year-old Norway spruce plants compared to untreated plants and plants that had been mechanically wounded on the lower stem. MeJA was not as effective as pesticide application in protecting the plants but did reduce spruce mortality significantly. Furthermore, the effect lasted for three growing seasons after treatment. We did not test lower concentrations of MeJA in the field. However, Chen et al. (2021) showed that 1-year-old Norway spruce plants sprayed with 10 mM MeJA had 78% less weevil damage than untreated control plants, suggesting that low MeJA concentrations may provide good protection. However, it is probably not realistic to expect MeJA treatment to protect Norway spruce plants as effectively as pesticide application (Cooper and Ton, 2022). Rather, the best strategy for using chemical priming stimuli in crop protection is probably to integrate these stimuli with other environmentally sound control strategies, such as physical stem protection. Implementing effective integrated pest management strategies against the pine weevil is of great importance, as it will reduce the reliance on environmentally harmful pesticides.

## Data availability statement

The raw data supporting the conclusions of this article will be made available by the authors, without undue reservation.

## Author contributions

PK: Conceptualization, Supervision, Methodology, Investigation, Formal analysis, Funding acquisition, Writing – original draft, Writing – review and editing. KK: Methodology, Investigation, Writing – review and editing. NH: Formal analysis, Writing – original draft, Writing – review and editing. MM: Formal analysis, Visualization, Funding acquisition, Writing – original draft, Writing – review and editing. All authors contributed to the article and approved the submitted version.

## Supplementary material

Supplementary Figure 1 Overview of experimental treatments, tree measurements, and number of replicates. Different chemicals were applied on the lower stem bark of 2-year-old Norway spruce plants: MeJA = methyl jasmonate, OxA = oxalic acid, SA = salicylic acid, Tween20 = water with 0.1% Tween20 (control). Different chemical concentrations were used in different sub-experiments (e.g., MeJA100 = 100 mM MeJA). Four weeks after treatment, plants were inoculated with the fungus *Endoconidiophora polonica* or left intact. Symptoms of fungal infection and different anatomical parameters were measured in the stem five weeks after fungal inoculation. Tree height growth was measured at the time of fungal inoculation and at harvest.

Supplementary Figure 2 Quantification of anatomical defense structures in the stem bark of 2-year-old Norway spruce plants 9 weeks after application of different chemical priming stimuli. Four weeks after application, three plants per treatment were inoculated with the fungus *Endoconidiophora polonica* and three plants were left intact. Upper panels: cross-sectional area of polyphenolic inclusions inside phloem parenchyma cells after treatment with high concentrations of oxalic acid (OxA; 200 mM), salicylic acid (SA; 500 mM) or methyl jasmonate (MeJA; 100 mM) (left panels) or different concentrations of MeJA (0, 5, 25, 50 or 100 mM with 0.1% Tween 20) (right panels). Control plants in the left panels were untreated or were treated with 0.1% Tween 20 (n = 3 + 3 = 6). Control plants in the right panels were treated with 0.1% Tween 20 (n = 3). Lower panels: cross-sectional area of cortical resin ducts (CD). Bars represent treatment group means ± 95% confidence interval of the mean. Points represent individual replicates. No significant differences were found between treatments (1-way ANOVA (~ treatment) followed by Tukey *post-hoc*, p < 0.05).
