# Supplementary figures and images for: Methyl jasmonate, salicylic acid, and oxalic acid affects growth, inducible defenses, and pine weevil resistance in Norway spruce
Source: Front Plant Sci. 2023 Jul 6;14:1155170. doi: 10.3389/fpls.2023.1155170 (PMC10357964; doi:10.3389/fpls.2023.1155170)

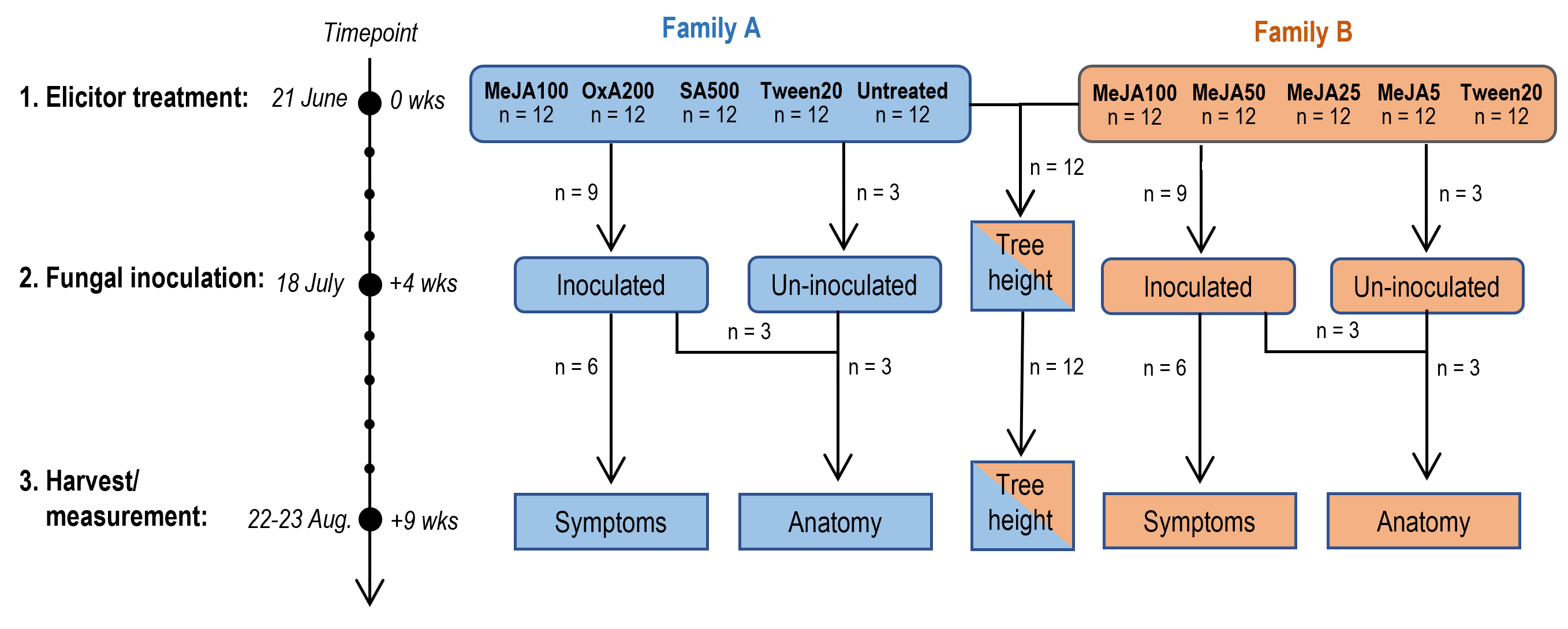

Supplement: Supplementary Figure 1 — Overview of experimental treatments, tree measurements, and number of replicates. Different chemicals were applied on the lower stem bark of 2-year-old Norway spruce plants: MeJA = methyl jasmonate, OxA = oxalic acid, SA = salicylic acid, Tween20 = water with 0.1% Tween20 (control). Different chemical concentrations were used in different sub-experiments (e.g., MeJA100 = 100 mM MeJA). Four weeks after treatment, plants were inoculated with the fungus Endoconidiophora polonica or left intact. Symptoms of fungal infection and different anatomical parameters were measured in the stem five weeks after fungal inoculation. Tree height growth was measured at the time of fungal inoculation and at harvest. [file Image_1.tif]

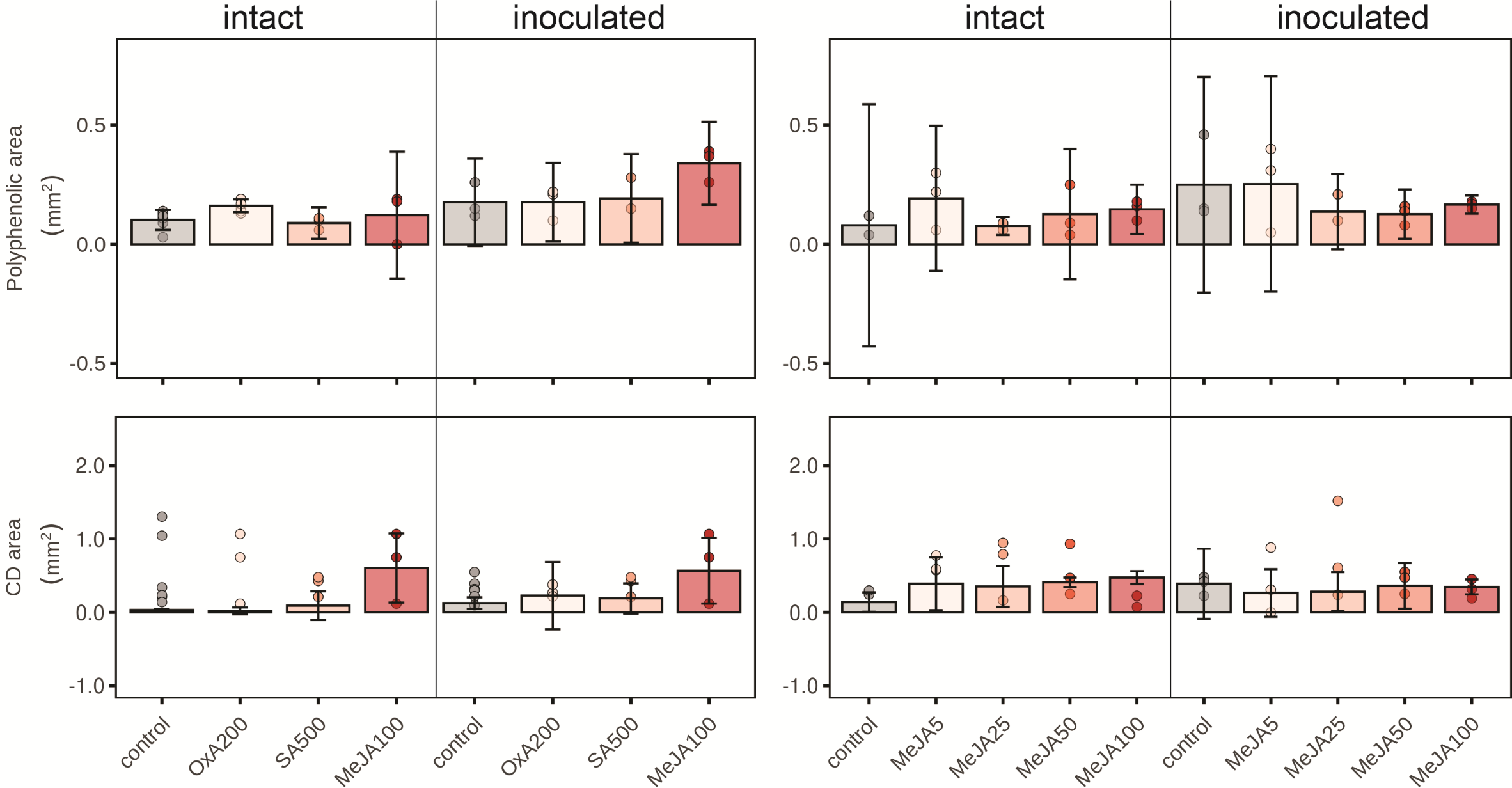

Supplement: Supplementary Figure 2 — Quantification of anatomical defense structures in the stem bark of 2-year-old Norway spruce plants 9 weeks after application of different chemical priming stimuli. Four weeks after application, three plants per treatment were inoculated with the fungus Endoconidiophora polonica and three plants were left intact. Upper panels: cross-sectional area of polyphenolic inclusions inside phloem parenchyma cells after treatment with high concentrations of oxalic acid (OxA; 200 mM), salicylic acid (SA; 500 mM) or methyl jasmonate (MeJA; 100 mM) (left panels) or different concentrations of MeJA (0, 5, 25, 50 or 100 mM with 0.1% Tween 20) (right panels). Control plants in the left panels were untreated or were treated with 0.1% Tween 20 (n = 3 + 3 = 6). Control plants in the right panels were treated with 0.1% Tween 20 (n = 3). Lower panels: cross-sectional area of cortical resin ducts (CD). Bars represent treatment group means ± 95% confidence interval of the mean. Points represent individual replicates. No significant differences were found between treatments (1-way ANOVA (~ treatment) followed by Tukey post-hoc, p < 0.05). [file Image_2.tif]
